# Significant Events Experienced by Psychiatric Patients With Personality Disorders in Inpatient Settings: A Qualitative Study and Implications for Clinical Management

**DOI:** 10.31083/AP44141

**Published:** 2025-06-23

**Authors:** Li Yang, Shu Yan, Shao-jiang Miao, Min Ma, Fan Yang, Bao-Liang Zhong

**Affiliations:** ^1^Department of Psychiatry, Wuhan Mental Health Center, 430012 Wuhan, Hubei, China; ^2^Department of Psychiatry, Wuhan Hospital for Psychotherapy, 430012 Wuhan, Hubei, China; ^3^Department of Psychological Counseling and Therapy Center, Wuhan Mental Health Center, 430012 Wuhan, Hubei, China; ^4^Department of Psychological Counseling and Therapy Center, Wuhan Hospital for Psychotherapy, 430012 Wuhan, Hubei, China

**Keywords:** hospitalized patients, significant events, personality disorders, qualitative research

## Abstract

**Background:**

Personality disorders are complex mental disorders characterized by interpersonal difficulties and are notoriously difficult to treat. Inpatient treatment offers patients the opportunity to establish therapeutic alliances, which can help alleviate their clinical dilemmas. However, there is currently a lack of research that takes the perspective of inpatients as the main subject. This study aims to delve into the significant events experienced by inpatients with personality disorders from their own perspective and explore their significance and impact on the individuals.

**Methods:**

Nine inpatients with personality disorders at different stages of hospitalization from a psychiatric specialty hospital were selected for semi-structured interviews. Grounded theory was used to analyze the data.

**Results:**

In the context of hospitalization, the significant events that patients experienced mainly include the ‘giving’ and empowerment by therapists, the contained and holding hospital environment, supportive relationships with peer patients, and the biopsychosocial impact of medication on patient perception and therapeutic engagement.

**Conclusion:**

Implicit ‘giving’ by therapists fosters empowerment and strengthens the therapeutic alliance, enhancing patient engagement and outcomes. The hospital environment offers a structured space for self-reflection and emotional recovery, while peer relationships promote growth. The combination of pharmacotherapy and psychotherapy stabilizes patients’ psychological states and improves receptivity to treatment. An integrated approach to these treatments is essential for optimizing patient outcomes.

## Main Points

- This study explores the experiences of inpatients with personality disorders from their own perspective.
- Empowerment by therapists, supportive hospital environment, and peer relationships are identified as key experiences.
- A structured hospital setting fosters emotional recovery and self-reflection.
- Combining pharmacotherapy and psychotherapy optimizes patient recovery and engagement.
- Grounded theory analysis provides new insights into the therapeutic impact of hospitalization.

## 1. Introduction

Personality disorders are often regarded as one of the most difficult mental illnesses to treat, primarily due to their complex pathological features and long-term course [1, 2, 3]. Additionally, personality disorders frequently co-occur with depressive disorders [4, 5, 6], post-traumatic stress disorder [7], anxiety disorders [8], and other Axis I mental illnesses [9, 10], which further exacerbates the impairment of social functioning in patients and severely hinders their normal functioning in daily life, professional settings, and interpersonal relationships. Particularly in terms of interpersonal relationships, individuals with personality disorders often face significant challenges, which hinder the formation of any effective therapeutic alliance in daily situations, and a therapeutic alliance is a prerequisite for successful treatment [11]. Therefore, it is particularly important to provide a stable and secure interpersonal therapeutic environment for patients with personality disorders. Inpatient psychotherapy can construct a comprehensive professional interpersonal therapeutic environment, thereby promoting the formation of effective therapeutic alliances. However, inpatient treatment for personality disorders is rarely reported in the literature. To the best of our knowledge, only a few studies have investigated its effectiveness, most of which have promising results [12, 13, 14]. Bartak *et al*. [15] compared the treatment outcomes of three psychological treatment environments for patients with cluster B personality disorders (outpatient, day hospital, and inpatient) and the findings identified the most effective treatment for psychopathological symptoms among patients receiving inpatient psychotherapy. Kraus *et al*. [16] also found that inpatient psychotherapy was effective for improving the level of patients’ personality functioning.

At present, research on the therapeutic efficacy of inpatients with personality disorders mainly focuses on the following aspects: using different diagnostic models to predict the effectiveness of outcomes [17], examining treatment effects through various intervention methods [18, 19], studying how to accurately and quickly identify patients with personality disorders and manage their hospitalization effectively from the perspective of clinical workers [20], and using objective measurement tools to measure and statistically test key factors in therapeutic efficacy [21]. Most of these studies set the outcomes as assessments made by clinicians. Few studies use subjective reports from patients to test the effectiveness of treatments. Nevertheless, the recovery of individuals with mental disorders is primarily a self-defining process. For instance, De Smet *et al*. [22] found that positive psychotherapeutic outcomes are not solely contingent on symptom reduction; rather, they are linked to the patient’s sense of empowerment and attainment of a new personal equilibrium, even if the individual continues to face ongoing struggles and conflicts during this progression. Therefore, investigating therapeutic factors in patients with personality disorders is a complex process that cannot be separated from the subjective feelings and experiences of patients. Understanding what plays a key role in the hospitalization process from the patient’s perspective is a necessary and less traveled path to exploring the path to healing in personality disorders.

Investigating the mechanisms of change in inpatient treatment for patients with personality disorders is crucial for continuously improving intervention outcomes. Greenberg used an event-based approach to study the process of change. He believed that an event consists of four components: the patient’s problem marker (e.g., a particular conflict), the therapist’s operation (intervention), the patient’s performance (response), and the immediate in-session outcome (e.g., the integration of conflictual tendencies or cognitive restructuring) [23]. Hill and Corbett [24] identified significant events in therapy as those that have a significant positive or negative impact on the patient. Identifying such significant events, i.e., moments that patients perceive as having a helpful impact, provides valuable data on the process of therapeutic change [25].

Significant events have also been used simultaneously as a research methodology to explore the identification of significant moments in the therapeutic process by individuals (mainly patients, but also therapists). Taking a therapeutic session as a unit, the therapeutic scene is revisited through audio or video recordings. The process of therapist-patient interaction is analyzed and interpreted from a microscopic point of view and different categories of significant events are obtained. However, such studies do not consider the whole dynamic process of therapy from a long-term perspective; particularly in inpatient settings, patients’ interaction patterns are much more complex than those in a simple individual therapeutic context, necessitating further in-depth research.

This study aims to adopt a ‘long lens’ approach to analyze the experiences of psychiatric inpatients with personality disorders by focusing on significant events. Specifically, it explores the critical events perceived by patients during hospitalization, examining their meaning and impact from the patients’ perspective. Through analyzing and reconstructing these events, the study seeks to identify key categories of significant events and potential therapeutic factors as understood from the viewpoint of the inpatients.

## 2. Methods

### 2.1 Inclusion and Exclusion Criteria

In this study, a purposive sample of nine inpatients was selected from Wuhan Mental Health Center, the largest psychiatric hospital in south-central China, between June to December 2019. The inclusion criteria were adult inpatients who had been diagnosed with both the Diagnostic and Statistical Manual of Mental Disorders, Fifth Edition (DSM-5) mood/anxiety disorders and personality disorders and were willing to participate in this study. In addition to traditional psychopharmacological treatments, these patients received individual psychotherapy twice a week and group psychotherapy once a day, with typical hospitalizations lasting around 3 months due to the complexity of their conditions. The exclusion criteria were patients in the acute phase of mood disorders and those with comorbid thought disorders. Of the nine patients, six were male and three were female, with an average age of 36 years (range: 24 to 58 years). The most common Axis I diagnosis was major depression (n = 5). The subtypes of personality disorders that the nine patients suffered from were borderline personality disorder (n = 3), paranoid personality disorder (n = 2), narcissistic personality disorder (n = 2), and avoidant personality disorder (n = 2). The relevant information is shown in Table 1.

**Table 1.** **Patients’ information**.

No.	Sex	Age	Diagnosis		Notes
		(years)	Axis I	Axis II	
P1	Male	28	Complex post-traumatic stress disorder, major depressive disorder, currently in a moderate episode	Borderline personality disorder	The patient reports having experienced complex trauma since childhood, including physical and emotional abuse. These experiences have led to long-term emotional instability, interpersonal relationship issues, and low self-esteem. The patient exhibits typical symptoms of borderline personality disorder, such as fear of abandonment, sudden anger, and self-harming behaviors. Recently, the patient’s depressive symptoms have worsened due to work-related stress and a breakup, leading to suicidal ideation, which prompted hospitalization for treatment. Hospitalized three times (this is the third time).
P2	Female	24	Bipolar disorder	Borderline personality disorder	The patient was sent to be adopted by another family at the age of 3 years and was taken back by their biological parents at the age of 8 years. The father is a person with emotional issues and is prone to emotional outbursts. The patient has always had problems with intimate relationships, being very quick to become infatuated with someone and just as quick to lose interest. Hospitalized on two separate occasions for a cumulative period longer than 1 year. Hospitalized for intimacy problems.
P3	Male	25	Major depressive disorder	Avoidant personality disorder	The patient began to experience symptoms of depression, feelings of oppression, and irritability half a year ago. He felt that people around him were deliberately targeting him and started to avoid social interactions. When the irritability became unbearable, the patient attempted suicide by jumping from a height. The patient has no close friends and, from a young age, was not allowed by their parents to act in a way that could be seen as coquettish; he was only permitted to do what his parents prescribed. He was constantly required to strive for perfection. First exposure to psychotherapy, second admission (previously hospitalized in a psychiatric unit with only medication).
P4	Male	28	Major depressive disorder, moderate severity, single episode	Narcissistic personality disorder	The patient presents with complaints of persistent feelings of sadness, loss of interest in previously enjoyed activities, and a significant decrease in self-worth, which he attributes to his recent divorce and business failure. First hospitalization and had been hospitalized for 2 months at the time of the interview.
P5	Male	29	Social phobia	Avoidant personality disorder	The patient feels extreme discomfort and anxiety in social situations, emotions that have affected him for many years but have significantly worsened in the past 6 months. He describes frequently experiencing an intense fear of being the center of attention, worrying about being judged or encountering embarrassment in social interactions, which leads him to avoid most social activities. Due to these social fears and avoidance behaviors, his career development has been limited, and family relationships have become strained. The patient expressed dissatisfaction with the current quality of life and concerns about the inability to socialize normally and form intimate relationships. He admitted to having suicidal thoughts but denied having any specific suicide plans or behaviors. First hospitalization, near discharge at the time of the interview, 3 months in hospital.
P6	Male	31	Major depressive disorder	Borderline personality disorder	The patient expressed his depressive mood that had progressively worsened in recent months, characterized by persistent sadness, inability to feel pleasure, and significant fatigue. In addition, the patient reported hallucinatory symptoms in which he heard voices discussing him and criticizing him. These voices were extremely disturbing to him and interfered with his sleep and daily functioning. He also faced chronic interpersonal challenges, including extreme fear of abandonment, frequent mood swings, and worry that he would not be able to meet the expectations of others, and often made impulsive decisions under pressure, such as quitting a job abruptly or severing a relationship with someone in a fit of anger. He also admitted to having suicidal thoughts but had not yet formulated a concrete plan. First hospitalization, late stage of hospitalization at the time of interview.
P7	Female	51	Major depressive disorder	Paranoid personality disorder	The patient is single and the eldest daughter in her family of origin. From a young age, she has taken on many of the roles of a mother within the family. However, she has always had a strained relationship with her younger siblings and particularly her father, which has also led to her inability to establish intimate relationships and has caused tension with her leadership at work. The current hospitalization was triggered by menopausal onset, which led to death anxiety and depressive mood. First hospitalization and had been hospitalized for more than 3 months at the time of the interview.
P8	Female	58	Major depressive disorder	Narcissistic personality disorder	The patient has been feeling persistent sadness and low mood over the past 3 years, especially in the last few months, where she feels she has lost her previous vitality and interest. She described a heavy sense of lethargy, making it difficult to complete daily chores and social activities. Additionally, the patient reported a decrease in appetite, sleep disturbances, and a sense of boredom or disinterest in activities she previously enjoyed. The patient also mentioned long-term interpersonal relationship issues, feeling that she is always misunderstood and is sensitive to criticism and feedback from others. She often feels competitive pressure in her relationships, worrying that she is not seen as successful and talented, and is concerned about her status in her work and social circles. She fears her achievements are no longer recognized and has difficulty accepting that she is no longer the center of attention. She is anxious about the changes that come with aging and struggles to adapt to life after retirement. One previous hospital stay of about 1 month, plus this hospitalization, totaling about 4 months.
P9	Male	50	Major depressive disorder	Paranoid personality disorder	The patient has been experiencing increasingly severe symptoms of depression over the past 6 months, including persistent sadness, a sense of hopelessness, and a significant decrease in his daily motivation. He finds it difficult to find meaning in life and has lost interest in his previous hobbies and activities, with notable appetite loss and sleep issues. He feels that he cannot trust colleagues and family, is often suspicious of others’ intentions, and believes others are talking about him or plotting against him behind his back. These paranoid beliefs have led to alienation from friends and family, increasing his feelings of isolation. He often feels misunderstood and disrespected and is worried about his health, reacting excessively to minor health issues, which further exacerbates his anxiety and depressive mood. The patient feels uneasy about life after retirement, fearing that the loss of his work identity could further intensify his depressive symptoms and social isolation. The patient was interviewed on two separate occasions, once at the beginning of the hospitalization, at about 1 month, and again at the end of the hospitalization, at 3 months (before discharge).

### 2.2 Procedures and Data Collection

The interview outline for this study is shown in Table 2. This outline was developed according to Greenberg’s theory of three dimensions of significant events, which was further informed by the Helpful Aspects of Therapy (HAT) scale [26] and the empirical taxonomy of helpful and nonhelpful events in brief counseling interviews [27]. In-depth, semi-structured interviews with patients were conducted in a one-on-one and face-to-face manner, with talk time ranging from 30 minutes to 60 minutes.

**Table 2.** **Interview outline**.

1. In your opinion, what is a significant event? (Connotation, extension)
2. During your hospitalization, what events were most significant to you personally? (The events can be positive or negative, and it can happen to different people such as therapists, nurses, psychiatrists, fellow patients, etc.)
3. Please carefully recall the situation at that time, how did the event happen? Who said or did what? How did each person respond to the other? How did the event end? Evaluate the extent to which the actions/responses of these people had an impact on you.
How much did this event help or hinder you? Please follow the scale below to assess this:
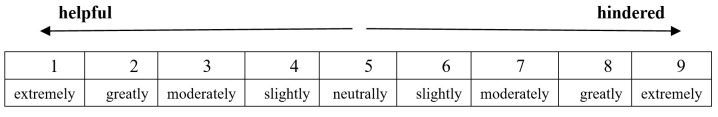
4. Describe your experience during the event:
A. How do you feel?
B. What were you thinking at the time?
C. What were you doing or trying to do?
5. Describe why this event was significant? What did you gain from it?
6. From now on, what are the strongest thoughts and feelings that this event has brought to your mind?
7. Describe what kind of changes and impacts may be brought to you in the future as a result of this event? Include both immediate impact (within 1 month) and subsequent impact (1 month later).
8. Apart from the above, is there anything else you would like to add?

Before the formal study, the ethics committee of Wuhan Mental Health Center approved the research proposal. The researcher and the participant engaged in a thorough informed consent discussion. Every step was executed following the patient’s consent and the signing of a written informed consent form. This included the interview process, audio recording of the interview, and the notation of key information including the patient’s non-verbal behaviors, insights derived, and associations made during the interview. It was mandatory that participants comprehended the purpose of the research and potential risks. Feedback to research subjects was also provided once the results were obtained, with consideration of the subjects’ perspectives and evaluations of the research findings. Furthermore, all individuals involved in the execution of this study consistently adhered to the ethical standards pertinent to the research.

After collecting the audio recordings, they were transcribed into verbatim scripts and coded by both researchers. Grounded Theory was used to code and analyze the information obtained by rooting in the data and working from the bottom up, and a log of coding and analysis was kept.

### 2.3 Data Sorting and Analysis

For data analysis, verbatim transcripts and memos were imported into Nvivo 11.0 (version 11.0, QSR International Pty Ltd, Melbourne, Victoria, Australia) for systematic coding using a three-stage Grounded Theory approach: Open Coding, Axial Coding, and Selective Coding [28]. The two coders, both experienced psychotherapists, first re-familiarized themselves with the interview context by reviewing the transcripts and audio recordings. Discrepancies in coding were resolved through discussion or, if unresolved, a third coder was consulted.

#### 2.3.1 Open Coding

In the initial open coding stage, conceptual categories were identified and their attributes and dimensions were defined, resulting in 242 free nodes.

#### 2.3.2 Axial Coding

In axial coding, connections between conceptual categories were analyzed, focusing on one category at a time to explore relationships and data linkages, producing a total of 25 tree nodes.

#### 2.3.3 Selective Coding

Finally, potential hypotheses based on the content of the data were continuously established and the data were iteratively compared with these hypotheses to generate a theory. The generated theory was then used to code the data again and establish a possible structural model for understanding the findings.

## 3. Results

In the process of hospitalization, patients continuously engage with therapists, fellow patients, and the hospital environment. In addition to this, medication exerts both physical and psychological effects on patients, as presented in Table 3. This stage represents a comprehensive, multi-layered interaction between the patient and the external object.

**Table 3.** **Themes: interactions with fellow patients, the hospital environment, and medication**.

Interactions with fellow patients	Daily entertainment and communication	
	Support among fellow patients	
	Conflicts between fellow patients	
	Emotional infection and influence of fellow patients	
Interaction with the hospital environment	Hospitals provide interpersonal opportunities	
	Hospitals offer patients a different experience	
	The hospital environment triggered a comparison	
	The isolating effect of the hospital environment	
	The changing hospital environment	
	The pressure of the hospital environment	
Interaction with medication	The medication relieved the symptoms	
	Perception of medication	Perceptions of the relationship between medication and psychotherapy
		Idealization and idealization breakdown of drug effects
	Psychological significance of drugs	

### 3.1 Therapist-Patient Interaction: Forms of Giving and Patient Responses

**Fig. 1.**
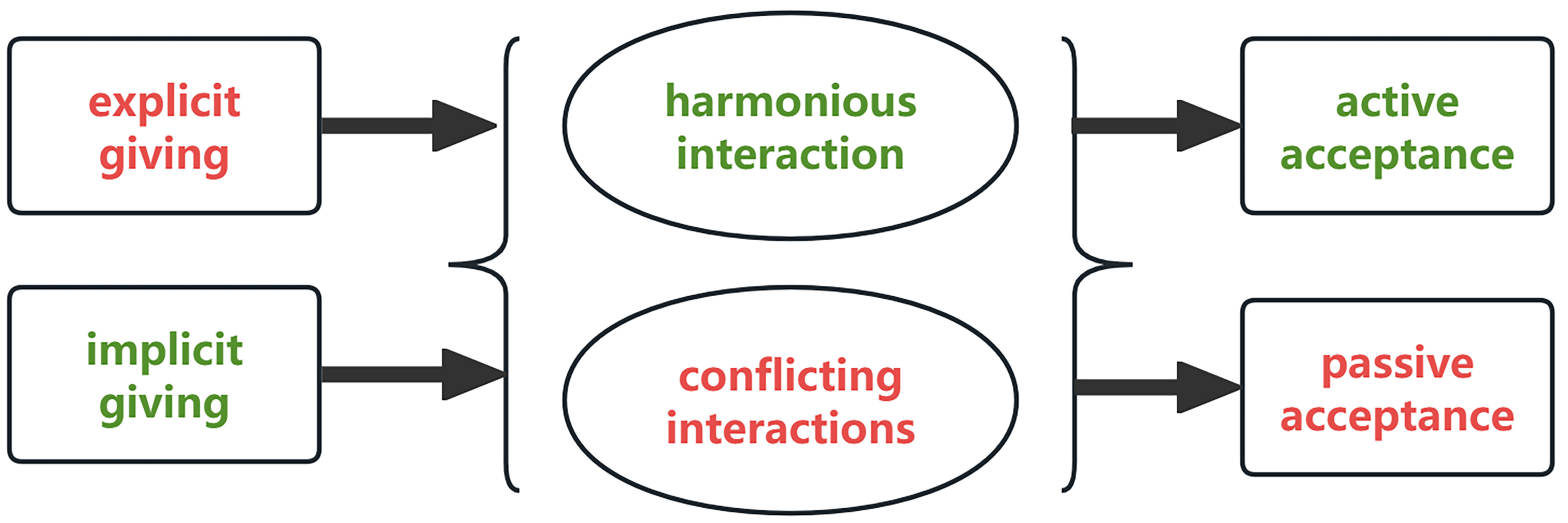
**Basic unit of therapist-patient interaction**.

The therapist-patient interaction in individual psychotherapy for patients with personality disorders is a central component in the hospitalization process, where the patient’s projections, conflicts, and relational dynamics manifest. This interaction creates a controlled environment, allowing patients to express, manage, and reflect on emotions and behaviors, fostering self-awareness. The therapeutic process can be divided into three main phases: the therapist’s ‘giving’, the interaction itself, and the patient’s response. The patient-therapist interaction presents the basic unitary process, as shown in Fig. 1.

The therapist’s ‘giving’ includes both ‘explicit’ and ‘implicit’ forms. Explicit giving involves direct verbal communication, such as explanations and guidance, helping patients to understand their symptoms and recognize underlying psychological issues. Implicit giving uses indirect methods, such as metaphors or non-verbal cues, to support self-awareness and provide empathic understanding. P1 mentioned that the therapist made ‘*a very rich body movement, just a gesture like this (making a gesture with both hands: placing both hands palm up in front of his abdomen and raising his hands upwards). It’s giving me a cue. This is important to me!*’ Patients often perceive implicit giving as more impactful, feeling acknowledged and supported in ways they may not have experienced in their daily lives. This creates a foundational support system, with the therapist sometimes becoming a significant figure in the patient’s life.

Patient responses to the therapist’s input can be active or passive. Active acceptance reflects a willingness to internalize insights and make changes, while passive acceptance may occur when patients comply due to the therapist’s authority, despite reservations. The interaction process itself may be harmonious or conflictual. Harmonious interactions occur when therapist and patient roles are naturally complementary, fostering trust and receptivity. Conflictual interactions arise from differing perspectives or unresolved transference, where unexpressed emotions are projected onto the therapist. These dynamic interactions reveal the complex layers of therapist-patient relationships, highlighting the critical role of relational attunement and patient receptivity in therapeutic effectiveness.

### 3.2 Peer Interactions and Mutual Support in the Hospital Environment

In the hospital environment, close daily interactions among patients serve as a primary form of interpersonal activity, both during and outside of treatment. Through shared activities—such as chatting, playing cards, singing, and participating in group therapy—patients not only fulfill their social needs but also learn to navigate interpersonal relationships, fostering personal growth. These interactions span cognitive, emotional, and behavioral dimensions, supporting patients’ psychological development within a structured community.

This peer interaction establishes a mutual support system where patients empathize and understand each other more readily, as noted by P2: ‘*In the hospital, you may have a similar situation with some of the same patients, and you will understand each other a little*’. Emotional contagion is also evident, where patients’ emotions impact each other within and beyond therapy sessions. P4 described how other patients’ experiences, such as personal or family issues, easily trigger their own emotional responses, reflecting a shared emotional vulnerability and reciprocal influence that enhances connection and empathy.

This dynamic of shared understanding, emotional resonance, and mutual support within patient interactions highlights the therapeutic value of peer relationships in a hospital setting, creating an environment conducive to emotional validation and psychological resilience.

### 3.3 The Hospital Environment as a Structured Space for Self-Reflection and Social Dynamics

The hospital environment serves as a structured, stable container that supports patients with a safe space for exploration, self-reflection, and socialization. It offers a unique interpersonal context where patients can observe and compare themselves with others who share similar struggles, fostering mutual support and empathy. This environment allows patients to feel less isolated, as expressed by P8, who noted that seeing others with similar challenges provided a sense of connection. Likewise, P2 highlighted the opportunity to engage with diverse perspectives, fostering a sense of belonging and facilitating self-awareness through relational dynamics and comparisons with peers. P2 also said ‘*I think I’ll get to know what a lot of people think. There could be opportunities to find out! … Everyone has their own unique way of getting along*’.

The structured nature of the hospital environment promotes exposure and reflection on personal issues, which can lead to insights and growth. For instance, patients experience a supportive yet controlled environment that contrasts with the outside world, bringing a sense of protection, belonging, and control. However, this contained setting also brings challenges, such as the temporary isolation from the external environment, which can be both relieving and restrictive. Long-term hospitalization may induce social detachment, as patients become increasingly aware of their ‘maladjustment’ when removed from everyday social contexts.

Finally, the hospital environment is marked by both stability and change, as patients navigate the departure of old friends and the arrival of new ones, which can disrupt the social dynamics they rely on. The regulated, sometimes coercive nature of hospital routines, such as assigned therapists and room arrangements, reinforces adaptation to structure and authority. As P3 and P1 described, they learned to adjust to aspects of the environment that were initially uncomfortable. Ultimately, the hospital becomes a ‘corrective environmental experience’, providing a balanced environment for restructuring psychological and relational patterns within a protective, transitional space between family and society.

### 3.4 The Biopsychosocial Impact of Medication on Patient Perception and Therapeutic Engagement

The interaction between patients and medication encompasses both biochemical and psychological dimensions. Medication provides direct symptomatic relief, notably improving sleep quality, emotional state, and reducing mental stress, as noted by P8, who described feeling like a ‘new person’ after receiving specific injections. While relief from somatic symptoms, especially improved sleep, is prominent, the psychological impact of medication evolves as patients’ perceptions of it shift alongside their engagement with psychotherapy.

Patients’ views on medication and psychotherapy often move from initial differentiation to eventual integration. Initially, patients may prioritize medication over psychotherapy, viewing them as separate treatments. For instance, P1 described initially focusing on medication’s calming effects without engaging deeply with psychotherapy. Over time, patients begin to see medication as foundational, supporting psychotherapy’s effectiveness. P3, for example, stated ‘*I feel that medication helps me to reach a certain state, and then the psychotherapy helps me to reach a better state. But without medicine as that foundation, psychotherapy could be in vain*’. He recognized that medication creates a baseline for mental stability, enabling deeper therapeutic work. This shift often involves a process where initial idealization of medication wanes as patients recognize its limitations, leading them to appreciate the combined role of both treatment modalities.

The psychological meaning of medication also relates closely to the therapeutic relationship, where attention and trust play key roles. Patients may express dissatisfaction or frustration if they feel that their medication needs and somatic discomforts are inadequately addressed by their therapists. As P1 described, unmet expectations regarding medication management led to feelings of mistrust, reflecting how the medication experience is intertwined with the patient’s perception of care and attunement within the therapeutic relationship. This complex interplay highlights how medication, beyond symptom relief, influences patient trust and engagement in therapy, shaping the broader therapeutic experience.

## 4. Discussion

### 4.1 The Role of the Therapist in the Interaction

#### 4.1.1 Explicit and Implicit Giving

The fundamental unit of therapist-patient interaction is the process of ‘giving and acceptance’. Compared with explicit giving, implicit giving is more readily embraced by the patient and is more likely to yield therapeutic benefits.

Dowell and Berman [29] found that therapist nonverbal behaviors, such as eye contact and trunk lean, could make the patient feel more empathy from the therapist. Yu *et al*. [30] showed that, compared with conventional interventions, therapists’ metaphorical interventions (implicit giving) can be more effective in relieving patients’ symptoms, patients are more likely to become cognitively involved and construct new life stories more creatively, and this kind of non-directive expression has a better long term memory effect, which can exert a more permanent therapeutic effect. This finding suggests that ‘implicit giving’ is a central influence within psychotherapeutic interactions. Furthermore, a higher degree of emotional involvement from the therapist is associated with reduced patient resistance and a strengthened therapeutic alliance.

#### 4.1.2 Authority and Empowerment

Compared with ‘explicit giving’, a therapist’s approach of ‘implicit giving’ is more readily accepted and internalized by patients on a subconscious level, fostering a sense of empowerment and enhancing the patient’s perceived control and agency over their psychological state. This sense of empowerment not only heightens patient satisfaction with the therapeutic process but also increases the likelihood of sustaining treatment benefits over time. Existing research underscores that supportive behavior and empowerment strategies by therapists are associated with high patient satisfaction and positive long-term prognosis [31]. Ryan and Deci [32] further emphasized the value of autonomy in therapy in Self-Determination Theory, stating that when patients feel a greater sense of control and engagement in the therapy process, their motivation and satisfaction increase accordingly.

Therapist-facilitated patient empowerment is thus recognized as a critical factor in the success of psychotherapy. The empowerment process not only encourages active patient engagement throughout the treatment but also contributes to the sustained effectiveness of therapeutic outcomes.

### 4.2 The Inpatient Setting — A Container of Containment

In individual therapy, the therapist serves as a ‘container’, holding the fragmented and confusing aspects of the patient’s experience and facilitating their clarification and re-identification. Within the context of hospitalization, however, the entire environment functions as a larger container, wherein the patient projects and navigates identifications with the various elements of this broader system. To support this process, it is essential to establish boundaries through rules, therapeutic contracts, and other structured settings, enabling the patient to gradually comprehend, adjust to, and derive a sense of security and authenticity from these parameters. More critically, the hospital environment—acting as a holding space—provides patients with a unique opportunity to confront and work through their symptoms, ultimately fostering the rediscovery of their true selves.

Fagin believes the inpatient ward represents the only constancy, a safe place able to contain and hold [33]. Gabay and Ben-Asher [34] further suggest that ‘in the context of hospitalization, patients anticipate that healthcare providers will hold and manage their pain, grasp its significance, and recognize the urgency of their crisis’. They emphasize that ‘providers who contain the emotional and cognitive experiences of patients contribute significantly to an enriched understanding of patients’ internal processes, emotions, and thought patterns [34]’. This act of containment fosters a therapeutic setting in which patients feel validated and supported, facilitating emotional recovery and self-awareness.

Jiang and Tong [35] propose that patients reconstruct their internal object relations during hospitalization, and that the conflicts that occurred in their family life are reenacted in their relationships with health care workers and fellow patients. The inpatient setting, however, offers a constructive context for these compulsive repetitions to be fully identified and explored therapeutically, providing patients with opportunities to address their conflicts. This therapeutic environment encourages patients to learn both to love and to accept love, fostering new or reconstructed means of finding contentment in their lives [35]. According to Tong [36], the therapeutic environment within hospital is structured to address patient needs through containing, structuring, involvement, support, and affirmation.

### 4.3 Patient Peer Relationships is a Way to Promote Patient’s Working Through

As patients 2 and 4 said, *‘the interpersonal relationship among fellow patients is the main interpersonal relationship during the hospitalization process’*. Xu *et al*. [37] referred to this interaction between patients as the ‘inter-patient relationship’ and stated that this inter-patient relationship has an impact on the mental state of patients in hospital through direct communication of different depths and indirect comparison and judgment (with patients).

Turner *et al*. [38] identified peer support as a powerful tool for empowerment, fostering a sense of hope, connection, and engagement. Their findings highlight how peer support contributes to increased self-acceptance, meaningful social roles and relationships, and strengthened self-determination. Additionally, self-awareness is often facilitated through both direct comparison and alternative learning with fellow patients. In a related study, Bloch *et al*. [39] examined therapeutic factors in group therapy, identifying key elements such as self-disclosure, interaction, acceptance (or group cohesiveness), insight, alternative learning, and altruistic behaviors as primary contributors to patient healing. These factors emerge naturally in the interactions among group members, illustrating the therapeutic potential embedded within a supportive group dynamic. During hospitalization, patients engage in various interactions within a relatively safe and therapeutic environment, where they are encouraged to communicate and share genuine experiences. This environment fosters mutual understanding, support, and acceptance among patients, which contributes to a cohesive, warm, and supportive atmosphere. Moreover, when the patient’s conflict or maladaptive patterns enact in the interaction of the patients’ peers, group therapy provides an opportunity for meaningful expression, where therapeutic intervention and interpretation can facilitate personal growth and behavioral change.

### 4.4 Pharmacotherapy and Psychotherapy: Synergy and Antagonism

Pharmacotherapy and psychotherapy engage with patients on distinct yet intersecting levels. Biochemically, medication directly interacts with the patient’s body, but also carries psychological meanings and effects, such as the placebo effect, and the expression of transference and counter-transference, often conveyed through the therapeutic use of drugs. Konstantinidou and Evans [40] found that in different therapeutic contexts, pharmacological and psychotherapeutic treatments for patients with personality disorders are frequently compartmentalized, with pharmacological and psychotherapeutic approaches standing in contrast to one another. This compartmentalization often creates a confrontation between the practical and psychological dimensions of treatment: which treatment is more effective? This situation can also potentially lead to patient splitting and enactment [40]. Norcross and Goldfried [41] argue that the effectiveness of medication often needs to be discussed in psychotherapy as well, and that the side effects of medication may become a focal point for fluctuations in the patient’s symptoms, which has an impact on the progress of psychotherapy. However, medication and psychotherapy may interact in surprising ways to bring about change. For example, medication may make patients more sensitive to psychotherapy, but may also contribute to the initiation and maintenance of new behaviors [41]. Beitman *et al*. [42] examined the perspectives of pharmacists, non-medical psychotherapists, and patients, analyzing this ‘triangle’ of pharmacotherapy and psychotherapy. They found that collaborative arrangements between psychotherapists and pharmacotherapists significantly contribute to the effective alleviation of patients’ symptoms. They propose that the basic principle in establishing this triangle is for each therapist to refrain from commenting on the other’s treatment approach. Ideally, the patient, along with both therapists, should meet to clarify the treatment plan collectively. Each therapist must initially explain their distinct role to minimize patient confusion and foster a strong therapeutic alliance. At the beginning of treatment, therapists should also articulate how psychotherapy and pharmacotherapy integrate to benefit the patient. Therefore, in-depth exploration of the combined effects of pharmacotherapy and psychotherapy from an integrative perspective is essential. Such an exploration would not only help to reveal how the two treatments complement each other at different levels, but also provide an important basis for developing more individualized and effective treatment plans.

## 5. Limitations and Implications

In this study, patients at different stages of hospitalization were interviewed to obtain different categories of significant events. However, because the selection of subjects was cross-sectional and there was no follow-up study of patients from admission to discharge, it is difficult to practically present the trend and dynamic process of the development of events as time advances.

Secondly, due to the limitations of the sample of this study, only nine samples were collected. The discussion on significant events for patients with personality disorders comorbid with Axis I disorders may have lacked typicality and specificity, and the saturation may have been insufficient. Additionally, the researcher’s own theoretical knowledge is limited, leading to insufficient ‘thick description’ of certain concepts during the interviews and analysis, which may have led to the shallow construction of the theory.

Third, in addition to the objective limitations of qualitative research itself, in the event study, the author found that there was a connection between the time period in which patients provided information and the time period in which they were interviewed. That is, patients tended to attribute events that occurred in the recent period as significant events. This, then, implies that for patients, the extents of significant events may have changed at different time periods. This further reflects the need for a follow-up study.

Therefore, in combination with the above three aspects, future studies should expand the sample size, conduct analysis in the interview process, and timely clarify the doubts in the interview to the patients, so as to promote the theory and reach saturation and sufficient depth. Additionally, a longitudinal design would mitigate the recency effect and further enrich the theoretical ‘flesh and blood’ by providing a more comprehensive understanding of event significance across time.

In summary, the hospital setting offers a therapeutic context for psychiatric inpatients with personality disorders serving as both an emotional and physical ‘container’. The interactions and conflicts of patients with different objects can be projected, validated, and resolved within the therapeutic context of hospitalization. These insights would facilitate the clinical management of patients with both Axis I and II disorders in inpatient settings.

## 6. Conclusion 

This study highlights the significance of therapeutic alliances and the hospital environment in the treatment of inpatients with personality disorders. The implicit ‘giving’ by therapists fosters empowerment, strengthens the therapeutic alliance, and improves patient engagement. The structured hospital environment supports self-reflection and emotional recovery, while relationships with peer patients contribute to personal growth. Additionally, the combined use of pharmacotherapy and psychotherapy stabilizes psychological states and enhances treatment receptivity. An integrated, holistic approach is crucial for optimizing patient outcomes.

## Data Availability

The qualitative data used in this study were collected from semi-structured interviews with nine patients with personality disorders. The interview guide is available upon request and includes the main themes and questions to ensure the reproducibility of the research. The interview recordings and transcripts will be stored on the researcher’s personal hard drive, accessible only to members of the research team. According to ethical requirements, participants may request the deletion of their data at any time after the study concludes. For further inquiries, please contact the authors for more information.
